# Ultra-Selective CMSMs Derived from Resorcinol-Formaldehyde Resin for CO_2_ Separation

**DOI:** 10.3390/membranes12090847

**Published:** 2022-08-30

**Authors:** Arash Rahimalimamaghani, David Alfredo Pacheco Tanaka, Margot A. Llosa Tanco, Maria Fernanda Neira D’Angelo, Fausto Gallucci

**Affiliations:** 1 Sustainable Process Engineering, Chemical Engineering and Chemistry, Eindhoven University of Technology, P.O. Box 513, 5600 MB Eindhoven, The Netherlands; 2 TECNALIA, Basque Research and Technology Alliance (BRTA), Mikeletegi Pasealekua 2, 20009 Donostia-San Sebastian, Spain; 3 Eindhoven Institute for Renewable Energy Systems (EIRES), Eindhoven University of Technology, P.O. Box 513, 5600 MB Eindhoven, The Netherlands

**Keywords:** carbon membranes, CO_2_ separation, post-combustion capture, supported carbon membranes

## Abstract

A resorcinol-formaldehyde precursor was synthesized to fabricate the CO_2_ selective Carbon Molecular Sieve Membranes (CMSMs) developed in this study. The degree of polymerization (DP) was analyzed via Gel Permeation Chromatography (GPC) and its effect on the CO_2_/N_2_ perm-selectivity and CO_2_ permeance was investigated. The membrane that was polymerized at 80 °C (named R80) was selected as the best performing CMSM after a preliminary test. The post treatment with oxidative atmosphere was performed to increase the CO_2_ permeance and CO_2_/N_2_ perm-selectivity on membrane R80. The gas permeation results and Pore Size Distribution (PSD) measurements via perm-porometry resulted in selecting the membrane with an 80 °C polymerization temperature, 100 min of post treatment in 6 bar pressure and 120 °C with an oxygen concentration of 10% (named R80T100) as the optimum for enhancing the performance of CMSMs. The 3D laser confocal microscopy results confirmed the reduction in the surface roughness in post treatment on CMSMs and the optimum timing of 100 min in the treatment. CMSM R80T100 exhibiting CO_2_/N_2_ ideal selectivity of 194 at 100 °C with a CO_2_ permeability of 4718 barrier was performed higher than Robeson’s upper bound limit for polymeric membranes and also the other CMSMs fabricated in this work.

## 1. Introduction

It is well accepted that the main contributor to the observed global warming is the greenhouse effect due to anthropogenic CO_2_ emissions resulting from the combustion of fossil fuels [1]. A short/medium-term solution to the problem is considered to involve capturing the CO_2_ directly from the source where the concentration of CO_2_ is high (i.e., flue gases of power plants, steel industry, cement industry, etc.) [2,3]. CO_2_ capture at an industrial scale is mainly performed through absorption processes using solvents [4], which, however, require high energy for desorption and regeneration (generally performed at higher temperatures), and suffer additional problems such as the loss of (often toxic) solvents.

Membrane separation technology is known for being compact, scalable, energy efficient and having small environmental footprint compared to competing technologies [5]. In recent years, due to the awareness about the consequences of the increasing CO_2_ levels in the atmosphere, more research has focused on developing membranes that selectively separate CO_2_ from gas streams in a typical post combustion CO_2_ capture configuration [6]. The developed membranes for CO_2_ should have high CO_2_ permeance and CO_2_/(other gas) perm-selectivity [7]. Despite the significant efforts made in regard to developing polymeric membranes, their main limitation for gas separation are: (a) they are rarely deployed in applications exceeding 100 °C due to their lack of long-term stability at high temperatures; (b) they are prone to plasticization (swelling and subsequent loss of permeation properties) due to the adsorption of gases (i.e., CO_2_) in the polymeric structure, especially at high pressures. Many high-performance polymers have thus been formulated but very few can be applied commercially, mainly due to plasticization; (c) they suffer chemical and biochemical degradation; and (d) they are subject to a trade-off between permeability and selectivity. Highly permeable membranes have low selectivity and vice versa, which is known as Robeson limit [8,9,10].

The properties of polymeric membranes can be improved by dispersing inorganic (i.e., zeolites, metals, metal oxides, carbon), metal-organic frameworks (MOFs) and nanofillers in the polymeric matrix, these composites are known as mix matrix membranes (MMMs). However, it is very difficult to disperse the nanofillers as they tend to sediment/agglomerate in the polymer chain matrix, restricting their wider application [11,12,13].

In contrast to polymer materials, inorganic membranes offer exceptional chemical, mechanical and thermal stabilities that increases their separation efficiency. Zeolite membranes can potentially separate gases due to their pore size at a molecular scale and due to their adsorption properties; however, zeolite membranes without defects are very difficult to prepare and they are expensive to produce [14,15,16]. Silica membranes can separate gases by the molecular sieving mechanism; however, they are not stable in gas streams containing water vapor at high temperatures [17].

Carbon molecular sieves membranes (CMSM) offer excellent stability at high temperatures, are chemically and biochemically resistant, do not suffer plasticization (due to the stability and strength of their aromatic structure based on a sp2 structure) and can surpass the Robeson limit of polymeric membranes [18,19]. CMSM are the product of the carbonization of thermosetting polymers in a non-oxidative environment and exhibit two transport mechanisms for gas separation: molecular sieving, where the gases smaller than the pores pass through the membrane, and adsorption diffusion, which depends on the adsorptive interaction of the molecules with the pores [20]. Understanding the relationship between the fabrication parameters and the CMSMs performance is considered the key factor to develop superior membranes that could be applied in industrial processes such as CO_2_ separation from flue gases, steel mill plant off gas, biogas upgrading, natural gas purification and steam methane reforming [21,22,23]. For instance, the pore size, pore size distribution and adsorption properties of the membranes can be modulated by changing the polymer precursor, degree of polymerization (DP or Xn of the polymer, the number of monomer units in the polymer chain), temperature and time of carbonization, addition of inorganic nanoparticles or metallic ions to the membranes structure [24].

In biogas upgrading, the necessity of a CO_2_ separation technology without losing a considerable amount of CH_4_ is considered the main challenge. Moreover, developed polymeric membranes such as Prism^®^, Carborex^®^ and Sepuran^®^ exhibit low permeances, which results in a higher surface area required. Lei et al. reported a decrease in the CO_2_ permeance of CMSMs by 23% by adding 1000 ppm n-heptane in the feed [25]. Recently, Yang et al. reported fluorinated carbon membranes that surpass the Robeson limit for CO_2_/N_2_ separation. However, due to high thickness and symmetric structure, the CO_2_ permeance is low and therefore these membranes are less suitable for industrial applications [26]. In addition, introducing H_2_S in the feed had a negative effect on CO_2_ permeance, but can result in an increase in CO_2_/CH_4_ selectivity. CMSM have been studied for biogas upgrading and the CO_2_/CH_4_ permeation properties of several of them have been shown to surpass the Robeson limit [27].

Developing CMSMs based on resorcinol-formaldehyde resin for gas separation has also been investigated in recent years. Rodrigues et al. reported CMSMs with a pore size distribution of around 0.6 nm for the membrane carbonized at 550 °C, with a general observation of a decrease in gas permeability with the increase in carbonization temperature [28]. Yoshimune et al. developed a highly mesoporous carbon membrane with a focus on the polymerization step and by changing the resorcinol-formaldehyde/catalyst ratio, producing membranes suitable for separating condensable gases such as CO_2_ and CH_4_ [29]. Dong et al. investigated the effect of quaternary ammonium addition to the resorcinol-formaldehyde CMSMs on the hydrophilicity and gas permeation properties. They reported the ability to control the pore size distribution by introducing tetra methylammonium bromide and tetra propylammonium bromide in the precursor synthesis [30].

The effect of the degree of polymerization in a resorcinol-formaldehyde polymer on the CO_2_ perm-selectivity, permeance and the effect of the membrane post treatment (etching the surface and pore walls in an oxidative atmosphere) on the CO_2_ permeance remains unknown in the open literature.

In this study, we have investigated the effect of the degree of polymerization on the performance of the supported CMSMs while also producing membranes with both high CO_2_ permeance and high perm-selectivity.

## 2. Materials and Methods

### 2.1. CMSMs Fabrication

Formaldehyde (37% VWR chemicals, USA), KOH pellets, N-methyl-2-pyrrolidone (NMP, 99.5%) and resorcinol for the synthesis of the membranes were purchased from Merck (Kenilworth, NJ, USA) and used as received without further purification. Asymmetric tubular porous alumina supports with an outer diameter (OD) of 10 mm and inner diameter (ID) of 7 mm, an external layer of alumina 100 nm in pore size and a length of 50 cm were supplied by Inopore GmbH (Veilsdorf, Germany).

One end of the porous supports was connected to the dense alumina tube and the other end was sealed with glass, as reported in previous papers [31]. The membranes were prepared by the dip, dry and carbonization method [32], as also reported in detail below. A resorcinol-formaldehyde oligomer was prepared and used to prepare the dipping solution, which contains formaldehyde and an acid catalyst; after dipping, the coated supports were heated under rotation to allow the on-site polymerization. The CMSMs were obtained after carbonization under a vacuum.

#### 2.1.1. Preparation of the Resorcinol-Formaldehyde Precursor

**Figure 1:**
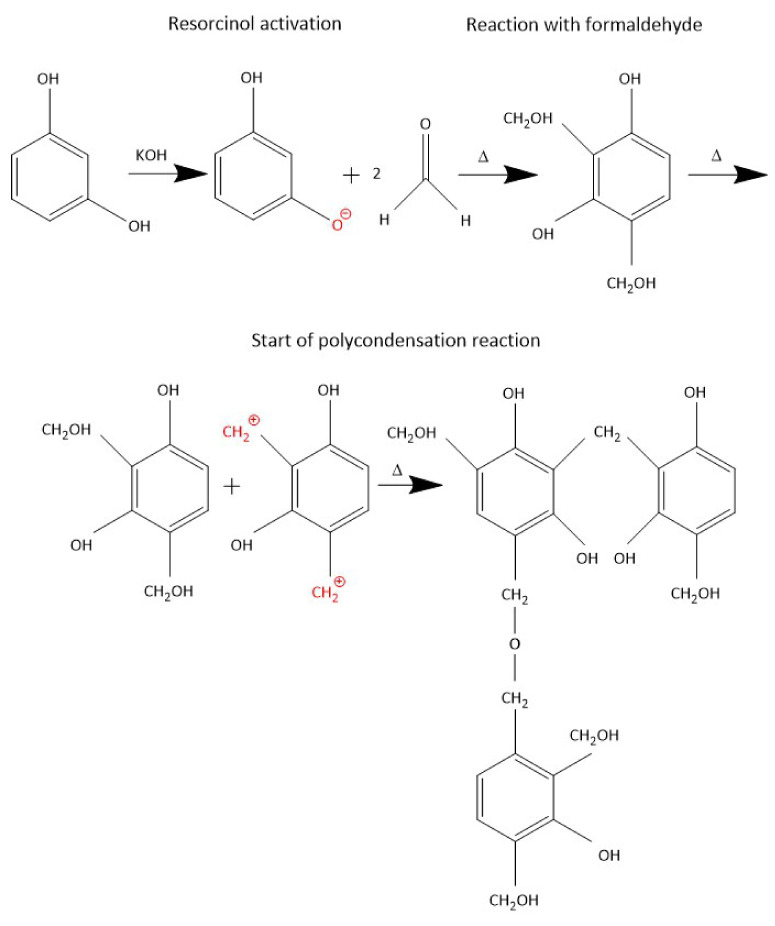
The schematic representation of resorcinol-formaldehyde oligomer synthesis (Δ is the heat of reaction).

The synthesis of the resorcinol-formaldehyde oligomers started by dissolving 80 g of resorcinol (0.726 mol) in 160 g of deionized (DI) water at 50 °C for 30 min with stirring in a round bottom 4 neck glass flask under reflux; 0.2 g of KOH was added to the solution. The temperature was increased to 90 °C and 118 g of formaldehyde solution (1.45 mol) was added to the mixture dropwise. The reaction was carried out for 3 h. Afterward, the oligomers were separated by centrifugation at 5 °C (4000 rpm for 15 min) and washed with deionized water three times. Finally, the oligomer was dried under a vacuum at 12 mbar and 40 °C for 24 h. The resulting oligomer powder was collected and kept in a gas tight vessel to be used for the preparation of a dipping solution. A schematic representation of precursor synthesis is illustrated by Figure 1 and the complete structure of the oligomer is indicated by Figure A3.

#### 2.1.2. Dipping Solution Preparation

Next, 16 g of the prepared resorcinol-formaldehyde oligomer was dissolved in 80 g of NMP using a high shear force mixer (Thinky ARE-250, Tokyo, Japan) at 1600 rpm for 30 min. The mixing cycle was repeated twice; between cycles, mixing was stopped for 15 min to prevent the solution from overheating. Next, 2.8 g of formaldehyde was introduced to the solution and mixed at 2000 rpm for 10 min. Then, 0.1 g of KOH was added and the solution mixed for 30 min at 1600 rpm. Finally, the whole solution was defoamed via the Thinky mixer for 30 min at 2000 rpm to remove any bubble.

#### 2.1.3. Dip Coating and Polymerization

The dip coating of the supports was carried out according to the previous work [33] with a downward speed of 10 mm/s, an upward speed of 5 mm/s, and a waiting time of 20 s. The coated supports were placed in an oven under N_2_ atmosphere for 24 h and kept rotating with a rotation speed of 30 rpm at various temperatures of 30, 60, 80 and 100 °C, corresponding to CMSMs named R30, R60, R80 and R100, respectively. The CMSMs are named by R followed by the temperature of polymerization (i.e., R80 is the resorcinol-formaldehyde resin polymerized on the porous support in an oven at 80 °C). After 24 h of rotation, the green membranes were transferred to a high temperature oven for carbonization.

#### 2.1.4. Carbonization

**Figure A1:**
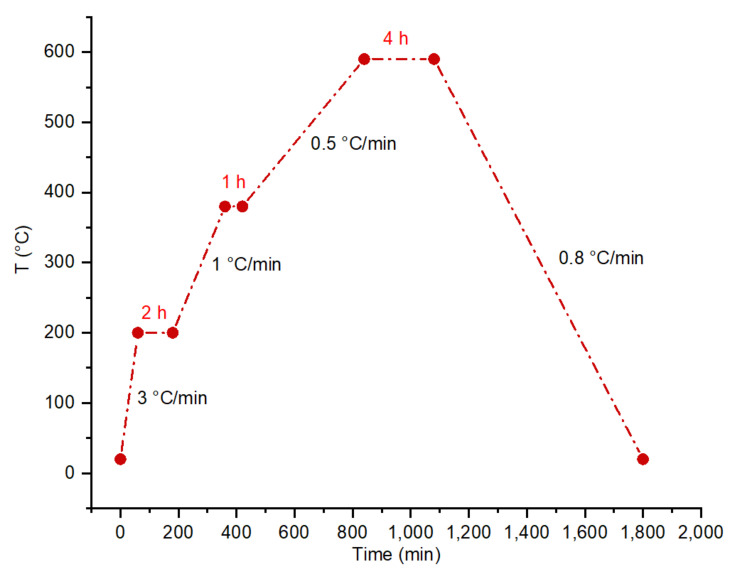
The carbonization protocol for CMSMs (Carbon Molecular Sieve Membranes).

The carbonization was carried out under a vacuum of 14 mbar in a three-zone controlled tubular oven (Nabertherm R 170/1000/1, Lilienthal, Germany), the temperature protocol is shown in Figure A1. After carbonization at 600 °C, the heaters were turned off and the oven was left to cool down to room temperature (Figure A1).

#### 2.1.5. Post Treatment

For a selected membrane with higher performance in terms of perm-selectivity and CO_2_ permeance (R80), two additional replicas were made. They were post treated with 10% oxygen at 6 bar operation pressure difference between permeate and retentate at 120 °C for 100 min (R80T100) and 150 min (R80T150).

### 2.2. CMSMs Characterization

#### 2.2.1. Gel Permeation Chromatography (GPC)

The analysis of polymerization degree (DP) as a function of polymerization temperature, was performed via GPC with SDV 500Å 5 µm column (Waters, model 2695, Milford, MA, USA) and THF as eluent. Support free polymer samples were digested for 48 h in THF solution and then injected to the GPC after filtration through a 2 µm filter.

#### 2.2.2. CHO Analysis

The self-supported CMSMs were fabricated by drying/polymerization of the dipping solution left in temperature ranges of 80 to 100 °C. After polymerization, the carbonization procedure was carried out identical to supported CMSMs and samples were used for the determination of the CHO composition using a Thermo Scientific, Flash smart- CHNS/O, Waltham, MA, USA analyzer. The calculation of the theoretical concentrations for the CHO atoms was based on the monomer indicated by Figure 1. Finally, the observed values from CHO analysis was compared with the calculated values to validate the polymer structure (Table 1).

#### 2.2.3. Scanning Electron Microscopy (SEM) Energy Dispersive X-ray Analyzer (EDX)

The samples were first sputter coated (Quorum, Q150RS, Sacramento, CA, USA) with Pt target for 20 s and a 30 mA current to prevent charge accumulation on the surface.

The supported CMSMs samples were analyzed via SEM-EDX (Phenom, ProX, Waltham, MA, USA) for C and O element weight percentages on the surface.

#### 2.2.4. D Laser Confocal Microscopy

To check the surface etching in the fabricated CMSMs after post treatment on the top selective layer, 3D laser confocal microscopy (VKX-3000, Keyence, Osaka, Japan) was performed on the membranes before and after the post treatment. CMSMs were analyzed without any special treatment at atmospheric pressure and air atmosphere.

#### 2.2.5. Perm-Porometry Tests

The pore size distribution (PSD) measurements in the fabricated CMSMs were performed by perm porometry using the equipment and method described in a previous study [33]. First, CMSMs were dried for 24 h at 350 °C under N_2_ at 5 bar pressure difference between retentate and permeate to remove any condensed water from the pores. Then, a membrane was cooled down to room temperature and helium was used as inert gas to measure the total permeance in the dry CMSM. Later, humidity was introduced stepwise with the injection of water to the helium stream to block the pores of the CMSM at 70 °C and 2 bar pressure difference. The pore size was calculated according to Kelvin’s equation, as indicated by Equation (1), where P, P_sat_, R, r, V_m_, γ and T are vapor pressure, saturated vapor pressure, universal gas constant, radius of the pore, molar volume of the liquid, vapor/liquid surface tension and temperature, respectively.

(1) $$\ln\frac{P}{P_{\mathrm{sat}}}=\frac{2{\gamma V}_{m}}{\mathrm{rRT}}$$

The percentage of the pores in that pore size was determined via a decrease in the permeate flowrate, as described in a previous study [33]. CMSMs were tested before and after the post treatment to realize the effect of post treatment timing and the polymerization temperature on the PSD in the fabricated CMSMs.

### 2.3. CMSMs Performance Tests

#### 2.3.1. Gas Permeation Setup

**Figure 2:**
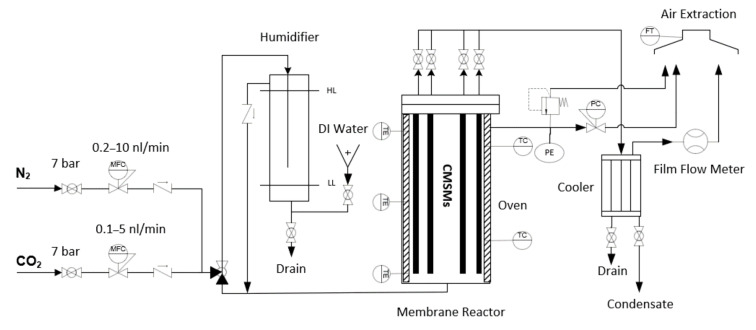
The gas permeation setup for testing 4 CMSMs (Carbon Molecular Sieve Membranes) with humidity function. MFC (Mass Flow Controller), HL (High Level), LL (Low Level), DI (Deionized), PC (Pressure Controller), FT (Flow Transmitter), TC (Thermocouple), PE (Pressure indicator), TE (Temperature indicator).

The CMSMs were tested in a membrane reactor in operational temperatures up to 350 °C and pressure difference of retentate and permeate up to 6 bar (Figure 2).

The gas permeation setup consists of a feeding section where CO_2_ and N_2_ flow rates were controlled. The gases can be fed to the reactor dry or humidified. A humidifier was used to stimulate the real conditions of post combustion, in which the flue gas is normally saturated with water. Next, the CMSMs were placed in a membrane reactor/separator with a capacity of four membranes, where the temperature was controlled by a two-zone oven. The selected membrane’s permeate flow was sent to a cooler to remove the water and the permeate flow rate was measured by a film flow meter (Horiba, Osaka, Japan). A system of valves was used to allow one membrane at a time to be analyzed while other membranes are still operational.

#### 2.3.2. Gas Permeation Tests

Due to the existing of hydrophilic sites in the CMSMs, before permeation, CMSMs were placed in the climate chamber with a relative humidity of 100% and a temperature of 20 °C for the aging process for a duration of one week. This is to make sure that all membranes are tested with the same initial status of humidification. Then, the CMSMs were placed in the membrane separator and N_2_ was injected with a flow rate of 2 L min^−1^. The operational pressure was set to 1 bar (g) and the temperature set to 45 °C with a heating ramp of 2 °C min^−1^. Then, the system was kept for 24 h in the same conditions to reach the stable performance of the permeated stream flow rate. Next, the permeation of N_2_ at pressure differences of 1, 2, 4 and 6 bar were measured at 45 °C with a feed flow rate of 5 L min^−1^ for each of the four CMSMs. In the next step, the pressure of the reactor was set to 0 barg to release all the N_2_ and CO_2_ was injected to the membrane reactor with a flow rate of 5 L min^−1^. The permeate flow rates were measured in the similar way than N_2_. Then, the reactor was heated up to the next operational temperature; after 30 min of stabilization, N_2_ and CO_2_ flow rates were measured as explained above. The flow rate was measured five times and the average was considered.

#### 2.3.3. Post Treatment with Oxygen

**Figure 3:**
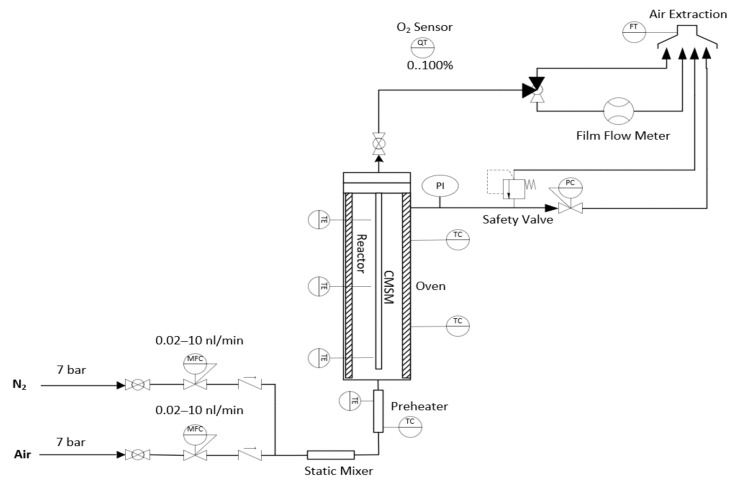
The membrane reactor for post treatment of CMSMs (Carbon Molecular Sieve Membranes) in an oxidative atmosphere. MFC (Mass Flow Controller), PC (Pressure Controller), FT (Flow Transmitter), TC (Thermocouple), PI (Pressure Indicator), TE (Temperature indicator).

The setup consists of a feeding section to feed nitrogen and air with the desired composition. Two new R80 CMSMs were prepared and humified; one of them was placed in a reactor inside an oven (Figure 3). The permeate stream flowrate was measured by a bubble flow meter (Horiba SF-VP, Osaka, Japan) and the pressure was regulated by a back pressure controller. First, N_2_ was fed to the reactor and the desired temperature of 120 °C and pressure difference of 6 bar between retentate and permeate were set. After reaching the permeation temperature and pressure, a mixture of N_2_ and air was fed to the reactor in such way that the oxygen molar concentration was 10% and treated for 100 min (R80T100). The other membrane was treated in the same way, except for the time of treatment that was 150 min and the other for 150 min (R80T150).

After post treatment, CMSMs were moved to a climate chamber for hydration and the gas permeation tests were carried out according to the aforementioned protocol.

## 3. Results

The molecular weight, C, H and O composition of the oligomer and the polymers polymerized for 24 h at different temperatures are listed in Table 1. The name of sample indicates the polymer related to the CMSM (for example R30-P, is the polymer of the membrane R30 which was polymerized at 30 °C). DP is calculated by dividing the measured polymer Mw found by the GPC method with the Mw of the monomer [34].

**Figure 4:**
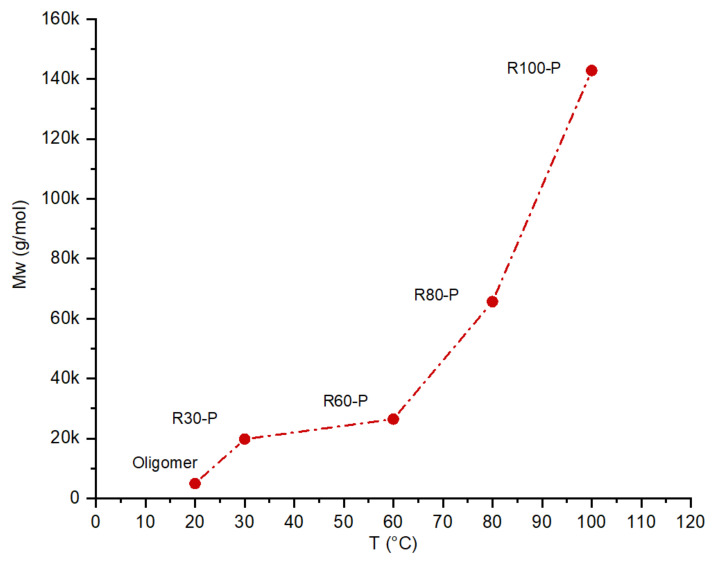
The effect of temperature of polymerization on the Mw (Molecular weight) of the resorcinol-formaldehyde polymer (time of polymerization, 24 h).

As indicated in Table 1, the composition of the polymer does not change with the DP; The experimental results of the composition and MW obtained by CHO analysis and GPC agree very well with the calculated values. Figure 4 indicates the effect of the polymerization temperature on the resorcinol-formaldehyde polymer’s MW. Due to the high activity of the oligomer, increasing the temperature from 20 °C (oligomer) to 30 °C has a significant effect on the MW (five times higher). This is related to the change in the reaction rate; as the temperature was increased, the reaction rate was also increased. Increasing the temperature from 30 °C to 60 °C, the MW increases only 1.3 times; as the temperatures increase to 80 and 100 °C, the degree of polymerization increases sharply.

**Figure 5:**
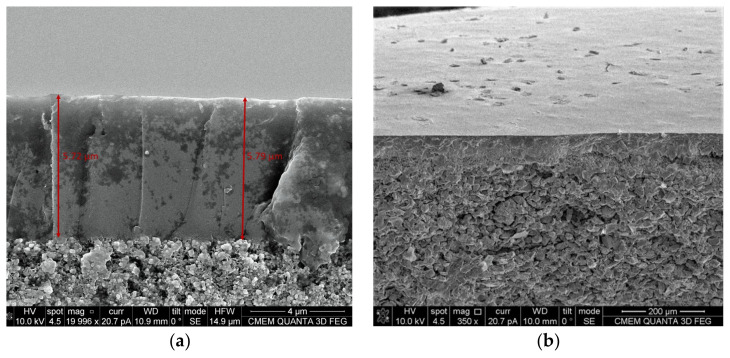
SEM images of R80 CMSMs (Carbon Molecular Sieve Membranes) magnified 20,000 (**a**) and 350 times (**b**).

The thickness of the supported CMSMs was measured by SEM, the average of several measurements at different cross sections of the membrane was used as an average thickness and was used for the calculation of the gas permeability of the membranes. Figure 5 shows the SEM images of the cross section in the CMSMs:

The changes in the selective layer thickness between the membranes were negligible and the average thickness for membrane R80 was measured at 5.7 µm with a standard deviation of 6%. As can be seen in Figure 5, the selective layer thickness is uniform and no defects are observed.

To analyze the composition difference between the surface and the bulk of the membrane, EDX was used to measure the surface composition in the fabricated CMSMs, and CHO was performed on the bulk samples; it should be considered that the values obtained by elemental analysis are more accurate than by EDX. Table 2 summarizes the results of the composition of the membranes for the membranes prepared with various temperatures of polymerization and for membrane R 80 after post-treatment.

For all the CMSMs, the oxygen content close to the surface (EDX) was higher than in the bulk (CHO elemental analysis). This could be explained by the reaction of the oxygen containing groups produced during carbonization with the pores of the membrane closer to the surface. Introducing post treatment with oxygen to membrane R80T100 and R80T150 resulted in a 55% and 95% increase in oxygen atoms on the surface, respectively, compared to the R80 membrane; as expected, with the increase in the time of treatment, the oxygen content increased. However, for the bulk of the membrane, the increase in O atoms was much smaller.

The 3D laser confocal microscopy technique was used to analyze the post-treatment on the morphology of the surface of the CMSMs by measuring the 3D surface structure and the average surface roughness.

**Figure 6:**
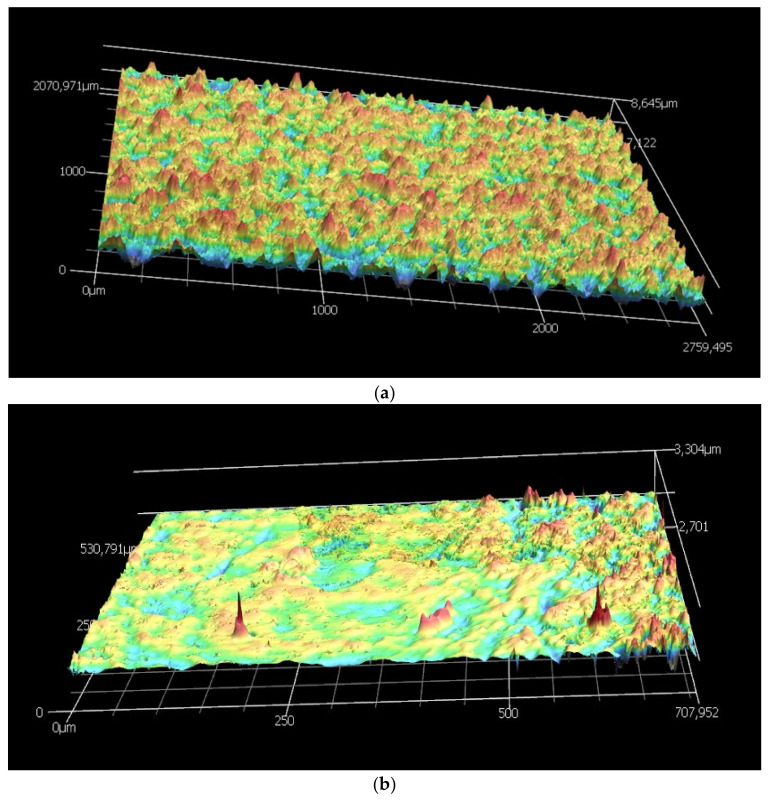
The 3D laser confocal scanning of R80T100 before (**a**) and after (**b**) the post treatment in oxidative atmosphere.

Figure 6 shows the comparison of the surface before and after the post treatment for membranes R80T100. It can be observed that the post-treatment oxidation reduces the surface roughness considerably. The roughness expressed in Ra and Rz values are listed in Table 3. The post treatment reduced the surface roughness for both 100 and 150 min. However, R80T100 exhibits a smoother surface (Ra, 39) compared to R80T150 (Ra, 164). This indicates that the longer oxidation of the surface in R80T150 results in more etching, creating higher surface roughness.

**Figure 7:**
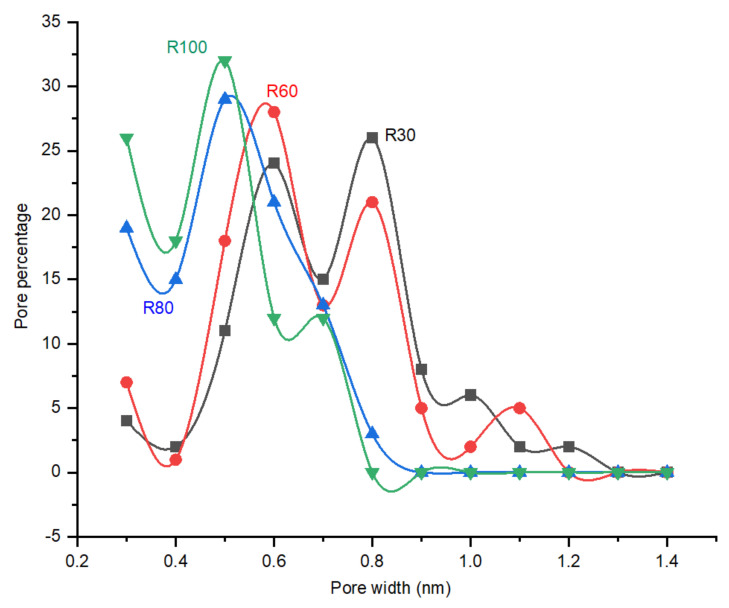
Effect of temperature on the PSD (Pore Size Distribution) of the CMSMs (Carbon Molecular Sieve Membranes) polymerized at various temperatures.

In this work, differently to previous reports, He was used instead of N_2_ as the inert gas to remove the water molecules condensed in the pores for the perm-porosimetry measurements. The kinetic diameter of He (0.260 nm) is smaller than N_2_ (0.364 nm), therefore smaller pores can be detected. The details of the method and setup are explained in detail in our previous work [33]. Figure 7 shows the measured pore size distribution of the CMSMs obtained at various polymerization temperatures. It can be observed that as the temperature of polymerization increases, the PSD is shifted to a smaller pore size due to the formation of a 3D network of aromatic rings linked by methylene and ether bridges by poly-condensation reactions of the aromatic and aliphatic alcohol groups in the polymer. After carbonization, the polymer results in a porous carbon matrix, in which the structure and pore size is directly related to the methylene and ether bridges in the polymer. The increase in linkage of aromatic rings results in smaller pores with a narrow pore size distribution. Smaller pores will increase the contribution of the molecular sieve transport mechanism in the CMSMs. The pores higher than 0.8 nm of R30 and R60 almost disappear when the temperature is increased to 80 and 100 °C. In membrane R80, the biggest detected pore size was measured at 0.8 nm, while for membrane R100, it was at 0.7 nm.

**Figure 8:**
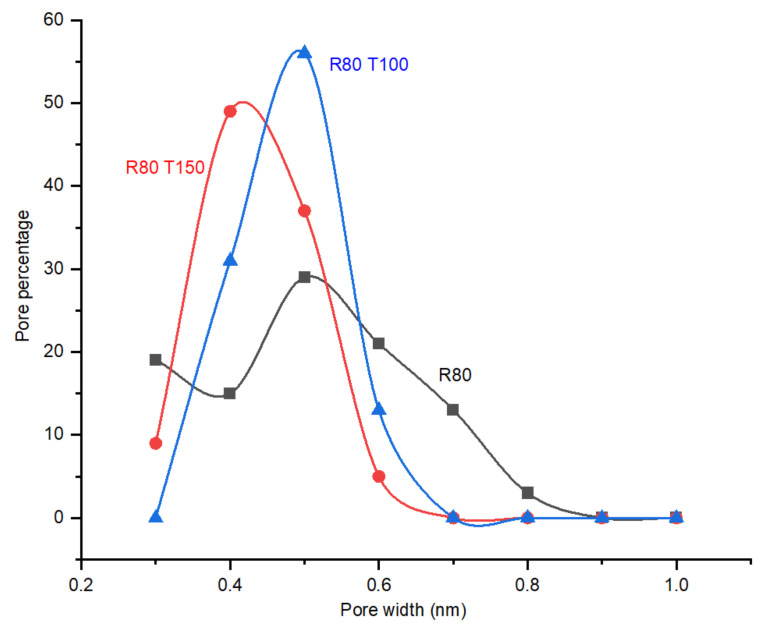
The effect of post treatment time (100 and 150 min) with 10% (molar) oxygen at 120 °C and 6 bar on the PSD (Pore Size Distribution) of the CMSM (Carbon Molecular Sieve Membrane) R80.

As illustrated in Figure 8, the post treatment with oxygen opens the pores smaller than 0.4 nm, resulting in the narrowing of the PSD. As the timing of the post treatment was increased from 100 min to 150 min, due to more etching of the pore walls with oxygen, the PSD was shifted to bigger pores in CMSM R80T150. The PSD width in the membrane with 150 min of post treatment remained the same as the membrane with 100 min of post treatment, suggesting the equal etching of the wall in the pores of the membrane between 100 and 150 min of post treatment. The optimum oxidation time seems to be close to 100 min for achieving the maximum performance in CO_2_ selective CMSMs.

## 4. Discussion

### 4.1. CMSMs Permeation Tests

The effect of pressure and temperature on the CO_2_ permeance and CO_2_/N_2_ ideal perm-selectivity of the membranes were studied in the range of 1–6 bar and 45–350 °C.

#### Effect of Temperature on CO_2_/N_2_ Ideal Selectivity and CO_2_ Permeance

**Figure 9:**
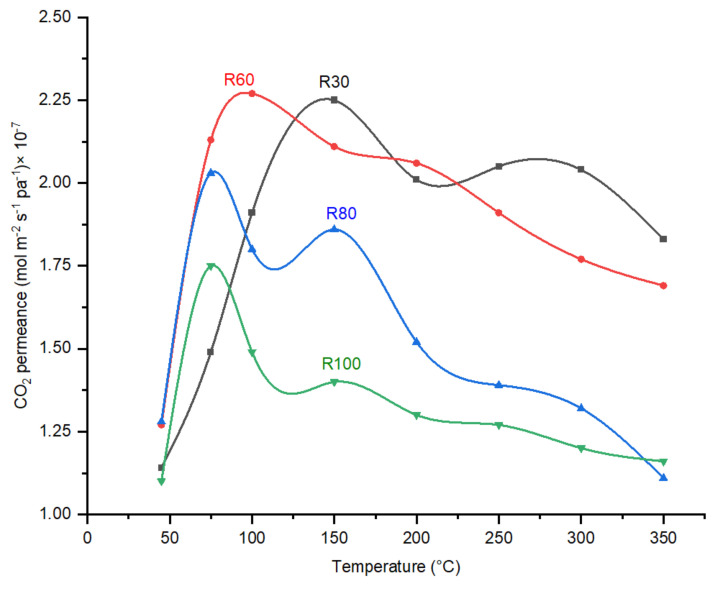
The effect of varying DP (Degree of Polymerization) on the CO_2_ permeances of CMSMs (Carbon Molecular Sieve Membranes) as a function of temperature at 6 bar.

As indicated in Figure 9, the CO_2_ permeance trend with increasing temperature follows the combination of molecular sieving and selective surface diffusion. CO_2_ permeance decreases as the membrane polymer (R100-P) increases DP. Shrinkage of the pores, according to the perm-porometry tests, sufficiently explains this behavior. Furthermore, due to the existence of adsorption sites in the CMSMs (0.6–0.8 nm, Figure 7), the membranes exhibit a decrease in CO_2_ permeance with the increase in temperature above 100 °C and the surface diffusion was reduced, which results in decrease in CO_2_ permeance.

**Figure 10:**
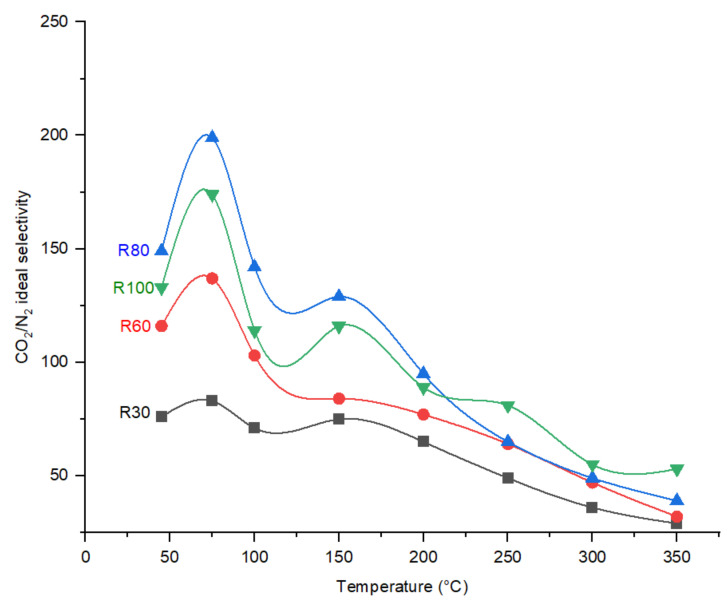
Effect of operational temperature CO_2_/N_2_ ideal perm-selectivity the CMSMs (Carbon Molecular Sieve Membranes) with varying the DP (Degree of Polymerization) in the polymer at 6 bar operational pressure.

The DP effect on the performance of CMSMs in terms of CO_2_/N_2_ ideal perm-selectivities as a function of permeation temperature is indicated in Figure 10.

In general, regarding the CO_2_/N_2_ ideal perm-selectivity, the fabricated carbon molecular sieve membranes indicate an increase from 45 °C to 75 °C, then a sharp decrease with increasing temperature. The existing selective sorption transport mechanism with an activation occurring around 75 °C reduces with a further increase in the operational temperature due to the decrease in adsorption at elevated temperatures. As the polymerization temperature increases from 30 °C to 80 °C, the reduction trend in CO_2_ permeance (Figure 9) shifts to lower permeation temperatures. The increase in ether and methylene bridges in the polymer with an increase in polymerization temperature results in the increase in functional groups after carbonization in the CMSMs. The increase in functional groups that act as sorption sites for CO_2_ molecules is considered a valid reason for this behavior. Increasing DP increases the CO_2_/N_2_ ideal selectivity in the fabricated membranes, which agrees with the perm-porometry results.

### 4.2. Effect of DP

By increasing the DP from membrane R30 to R100 at 75 °C, the CO_2_/N_2_ ideal selectivity reaches its maximum in membrane R80 and decreases as the DP further increases in membrane R100. The trend in CO_2_/N_2_ ideal selectivity for the fabricated CMSMs are explained by the PSD measured by perm-porometry which is also performed at 75 °C. CMSM R80 exhibited the maximum CO_2_/N_2_ ideal perm-selectivity while containing the narrowest pore size distribution compared to membranes R30, R60 and R100.

### 4.3. Effect of Post Treatment

**Figure A2:**
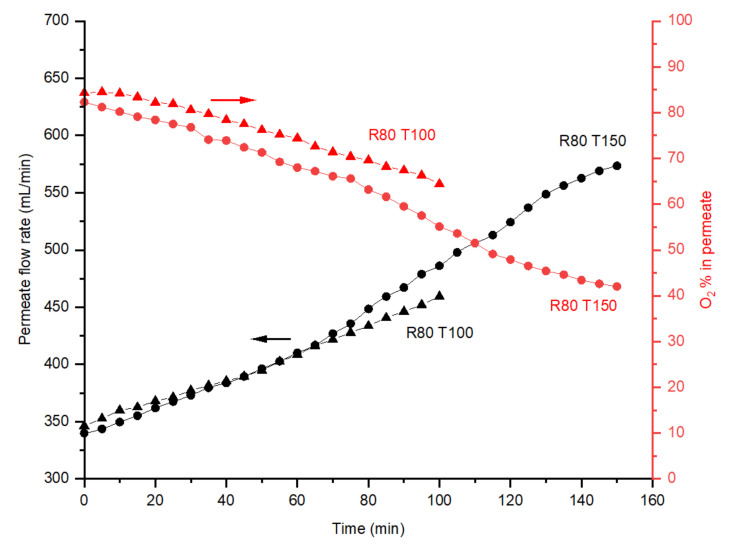
The effect of post treatment of CMSMs (Carbon Molecular Sieve Membranes) in the permeate flow rate (SD 4%) and oxygen concentration in permeate, measured by GC (Gas Chromatograph) that are treated at 120 °C and 6 bar with 10% oxygen in the feed stream.

**Figure A3:**
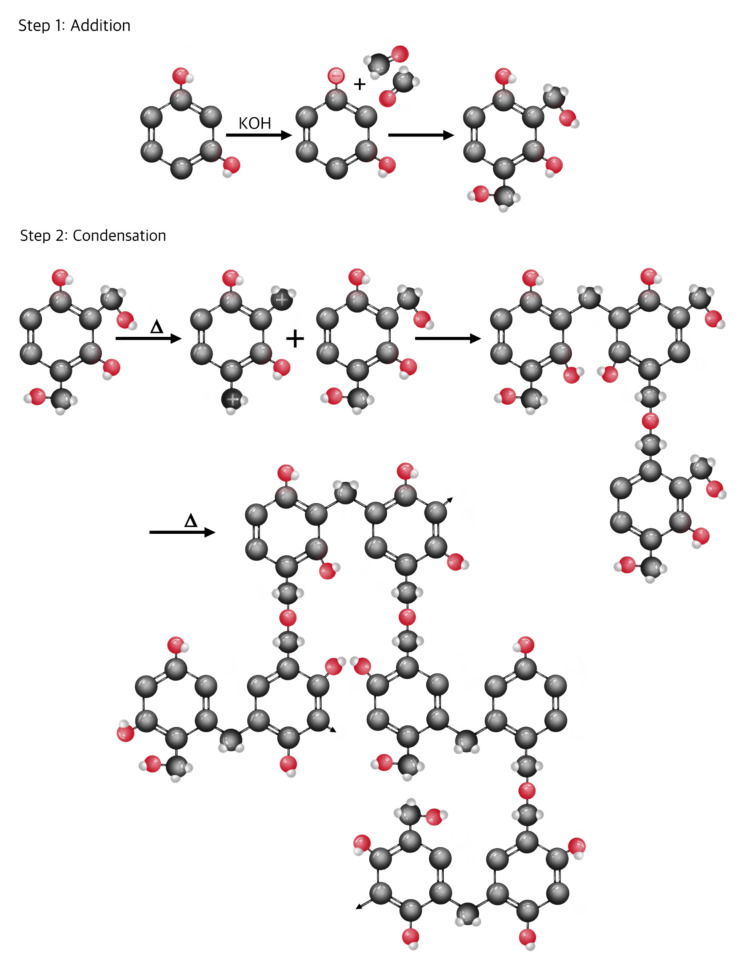
The schematic of polycondensation reaction of resorcinol with formaldehyde in the presence of KOH as catalyst.

CMSMs R80T100 and R80T150 were produced from identical R80 CMSMs with post treatment in 10% oxygen concentration for a duration of 100 and 150 min, respectively. The permeate stream flowrate through the membranes were logged every 5 min and the composition of the permeate was analyzed with a gas chromatograph (GC, Agilent 490). The oxygen concentration in the permeate was reduced as the etching was continued in both R80T100 and R80T150 (Figure A2).

Both R80T100 and R80T150 CMSMs exhibited identical behavior with the increase in the permeate flowrate until 75 min from the start of oxygen injection. CMSM R80T150 exhibited a sharp increase in the permeate flow rate from a time interval of 75 to 130 min, most probably due to the opening of the dead-end pores or widening of sub 0.4 nm pores, which is in line with the results of perm-porometry.

**Figure 11:**
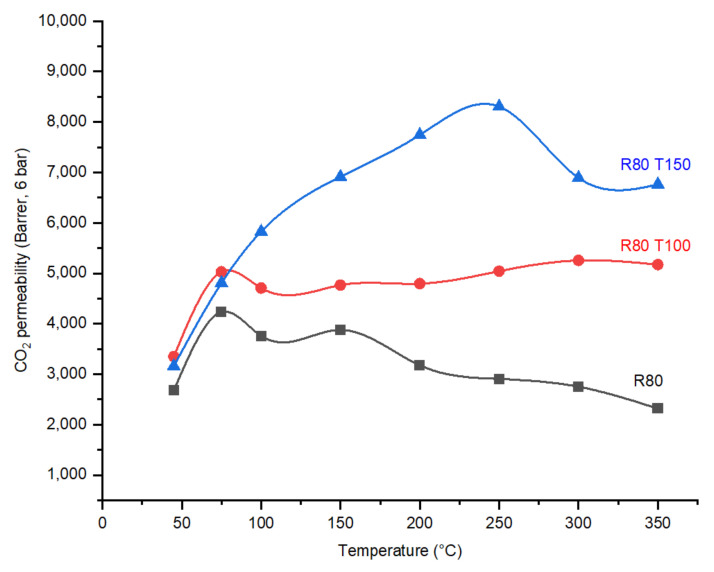
The effect of post treatment with oxygen on the CO_2_ permeabilities of CMSMs (Carbon Molecular Sieve Membranes) as a function of temperature.

As expected, the effect of post treatment on membranes R80T100 and R80T150 resulted in an increase in CO_2_ permeability due to the opening of the pores mainly below the kinetic diameter of the CO_2_ (Figure 11), which is approved by the perm-porometry results.

**Figure 12:**
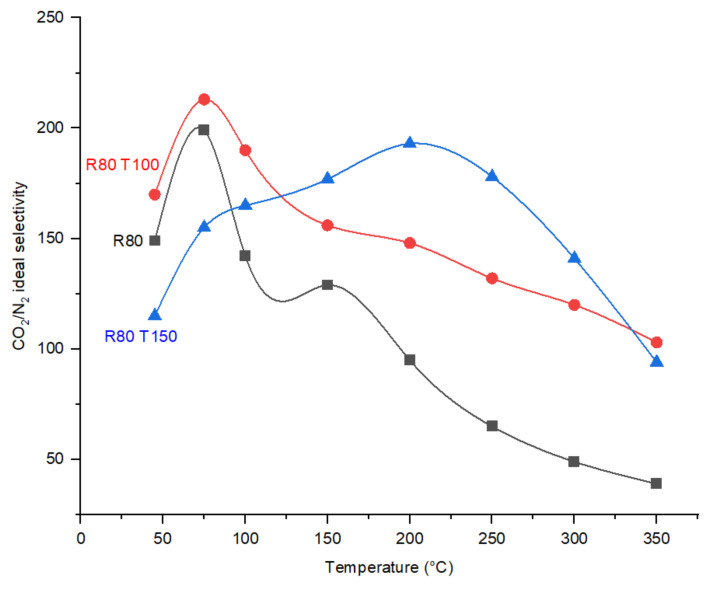
Effect of post treatment with oxygen on the CO_2_/N_2_ ideal perm-selectivities of CMSMs (Carbon Molecular Sieve Membranes) as a function of temperature.

In the fabricated membranes with the same DP, R80, R80T100 and R80T150, the highest CO_2_/N_2_ ideal selectivity value at 75 °C was exhibited by membrane R80T100, which is 213 at 6 bar operational pressure. When comparing the PSD in the CMSMs with the same DP, R80T100 contains the larger number of pores in the pore size of 0.4 nm, which may explain the higher selectivity. Membrane R80T150 reaches the maximum value of CO_2_/N_2_ ideal perm-selectivity in an operational temperature in the range 150–300 °C compared to other CMSMs (Figure 12). The creation of additional functional groups containing oxygen on the pore walls during the post treatment of R80T150 explains the higher activation energy of CO_2_ transport in the porous structure and the higher interaction of membrane R80T150 with CO_2_. The bulk (CHO) and surface (EDX) elemental analyses (Table 2) confirm the increase in oxygen and hydrogen concentration in both R80T100 and R80O150 compared to the R80 membrane.

**Figure 13:**
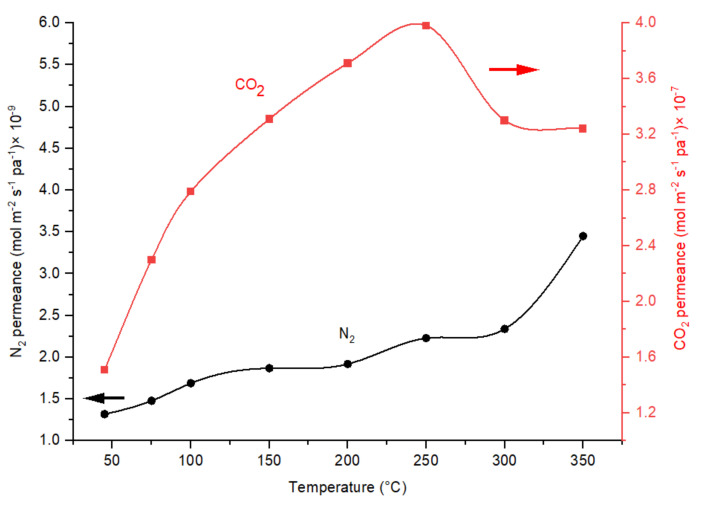
Effect of temperature on the CO_2_ and N_2_ permeances in CMSMs (Carbon Molecular Sieve Membrane) R80T150 in operational pressure of 6 bar.

The comparison of N_2_ and CO_2_ permeances as a function of temperature in Figure 13, indicates the dominance of the change in CO_2_ permeance in the determination of CO_2_/N_2_ ideal perm-selectivity. N_2_ permeance gradually increases with the increase in the permeation temperature due to the molecular sieve transport mechanism in the CMSM for N_2_, and not the interaction of N_2_ molecules with the pores. In CMSM R80T150 the permeance of CO_2_ was increased sharply from 50 to 250 °C, while N_2_ permeance was almost constant (Figure 13).

### 4.4. Effect of Pressure

**Figure 14:**
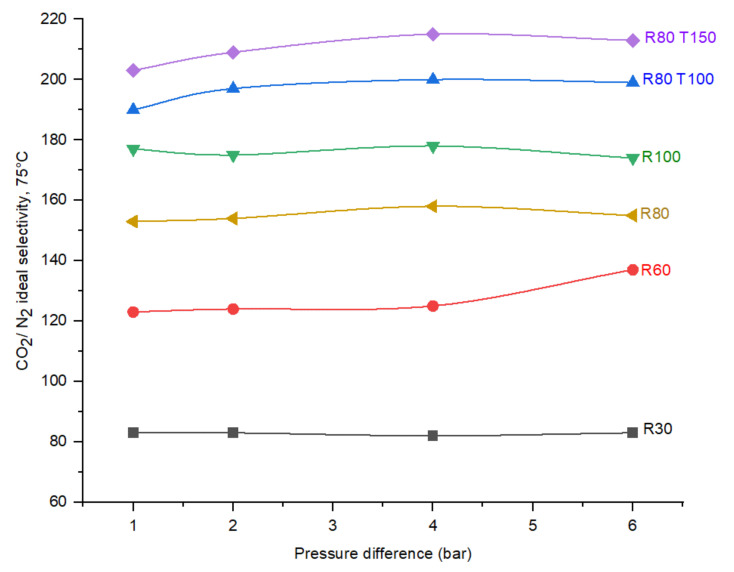
Effect of operational pressure on the CO_2_/N_2_ ideal selectivity in the CMSMs (Carbon Molecular Sieve Membranes) with varying the DP (Degree of Polymerization) in the polymer and applied post treatment at permeation temperature of 75 °C.

To realize the effect of pressure in CO_2_/N_2_ ideal perm-selectivity in CMSMs, the permeation tests were carried out at 1, 2, 4, and 6 bar. Figure 14 indicates the performance of the membranes at multiple pressures and 75 °C permeation temperature:

In all the fabricated membranes, according to Figure 14, due to the pure gas injection and nonexciting concentration polarization on the surface of the CMSMs, the effect of pressure on the CO_2_/N_2_ ideal selectivity is negligible.

### 4.5. Comparison of Membranes’ Performance

**Figure 15:**
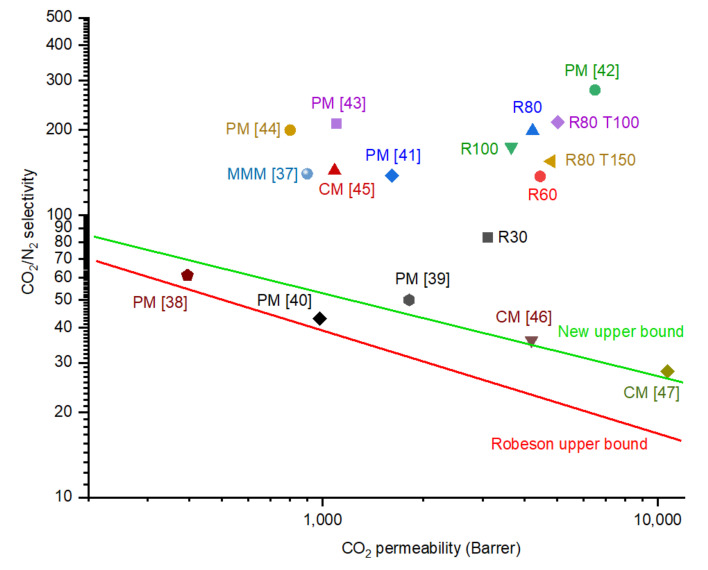
Comparison of performance of CMSMs (Carbon Molecular Sieve Membranes) with varying the DP (Degree of Polymerization) in the polymer and applied post treatment at permeation temperature of 75 °C and 6 bar pressure difference. The comparison based on the Robson’s upper bound limit of polymeric membranes, recent Polymeric Membranes (PM), Mixed Matrix Membranes (MMM) and Carbon Membranes (CM) [37,38,39,40,41,42,43,44,45,46,47].

Fabricated CMSMs performance is compared to the upper bound limits of polymeric membranes from 2008 [35] and 2019 [36] in Figure 15. All the CMSMs performed beyond the upper bound limits and membrane R80T100 was selected as the best performing membrane, with the highest value for the CO_2_/N_2_ selectivity (213) and CO_2_ permeability (5030 barrer) at operational pressure and temperature of 6 bar and 75 °C, respectively. The increase in CO_2_ permeability will make the CO_2_ selective CMSMs require less permeating surface area; meanwhile, the higher CO_2_/N_2_ selectivity will result in a one stage separation process to reach the high purity of CO_2_ in the permeate, which reduces the capital and operational costs. The stable performance of R80T100 at elevated temperatures, surpassing the upper bound limits (Figure 15), increases the potential of CMSMs synthesized from the Resorcinol-formaldehyde precursor to be applied in CO_2_ separation industrial processes.

## 5. Conclusions

CMSMs were fabricated with a precursor synthesized with resorcinol-formaldehyde polymerization on asymmetric alumina supports. The performance of the membranes was tested in the temperature range of 45–350 °C and the pressure difference of 1–6 bar. The membranes exhibited surface diffusion and a molecular sieving transport mechanism. The DP of the polymer was investigated as a function of polymerization temperature and the membrane with a polymer synthesized at 80 °C exhibited the best performance in terms of CO_2_/N_2_ ideal selectivity and CO_2_ permeability. The increase in the polycondensation in the polymer resulted in shifting the PSD to a smaller and narrower pore size. The post treatment with 10% oxygen at 120 °C was performed for a duration of 100 and 150 min. In both membranes, the post treatment increased the performance of the membrane and the combination of SEM-EDX with CHO elemental analysis resulted in a high concentration of oxygen atoms on the surface of the membrane. The 3D confocal microscopy results confirmed the etching effect of post treatment with oxygen on the membrane surface with the measuring of average roughness. CMSM R80T150 exhibited a superior performance at 250 °C, which could make it a potential candidate for high temperature CO_2_ separation processes such as membrane reactors. All the fabricated CMSMs performed higher than Robeson’s upper bound limit of polymeric membranes.

## Figures and Tables

**Table 1:** Gel Permeation Chromatography (GPC) results of Degree of Polymerization (DP) and Molecular weight (Mw) of resorcinol-formaldehyde polymer.

Sample (Polymer)	C			H			O			MW		DP
	Cal	Obs *	Cal	Cal	Obs *		Cal	Obs *		Cal	Obs **	
	(%)		#	(%)		#	(%)		#	(g mol^−1^)		##
Oligomer	61.5	61.9	250	5.7	5.5	280	32.8	32.6	100	4880	4939	10
R30-P	61.4	62.2	1025	5.7	5.8	1148	32.9	32	410	20,008	19,766	41
R60-P	61.5	62.4	1350	5.7	5.6	1512	32.8	32	540	26,352	26,420	54
R80-P	61.5	61.9	3375	5.7	5.6	3780	32.8	32.5	1350	65,880	65,640	135
R100-P	61.5	62	7325	5.7	5.8	8204	32.8	32.2	2930	142,984	142,790	293

Results obtained from: * CHO micro analysis and ** gel permeation chromatography. # number of atoms in the sample. ## number of monomers in the polymer.

**Table 2:** Composition of the CMSM prepared at various polymerization temperatures.

Membrane	SEM-EDX (wt%)		Organic Elemental Analysis (wt%)		
	C	O	C	O	H
R30	90.6	6.3	92.1	4.9	3
R60	92.7	5.7	94.6	3.1	2.3
R80	94.2	3.8	96.1	2.6	1.3
R100	96.5	3.2	97.2	2.1	0.7
R80T100	91.1	5.9	95.9	2.7	1.4
R80T150	88.9	7.4	95.3	2.9	1.8

**Table 3:** Post treatment effect on the surface roughness in CMSMs (Carbon Molecular Sieve Membranes).

Membrane	Before Post Treatment (nm)		After Post Treatment (nm)	
	Ra	Rz	Ra	Rz
R80T100	1347	8843	39	399
R80T150	1266	8690	164	1099

## Data Availability

Data will be made available upon request.
